# Sedentary Behaviour and Biomarkers for Cardiovascular Disease and Diabetes in Mid-Life: The Role of Television-Viewing and Sitting at Work

**DOI:** 10.1371/journal.pone.0031132

**Published:** 2012-02-09

**Authors:** Snehal M. Pinto Pereira, Myung Ki, Chris Power

**Affiliations:** MRC Centre of Epidemiology for Child Health, UCL Institute of Child Health, London, United Kingdom; Innsbruck Medical University, Austria

## Abstract

**Background:**

Knowledge of sedentary behaviour associations with health has relied mainly on television-viewing as a proxy and studies with other measures are less common. To clarify whether sedentary behaviour is associated with disease-risk, we examined associations for television-viewing and sitting at work.

**Methods:**

Using the 1958 British birth cohort (n = 7660), we analysed cross-sectional associations between television-viewing and work sitting (four categories, 0–1 to ≥3 h/d) with total, high-density lipoprotein (HDL) and low-density lipoprotein (LDL)-cholesterol, triglycerides, blood pressure, glycated haemoglobin, fibrinogen, C-reactive protein, hypertension and metabolic syndrome at 45 y. We adjusted for lifestyle and socio-demographic factors and assessed mediation of associations by body mass index (BMI) and diet. We also assessed whether the sedentary indicators are related similarly to factors linked to disease-risk.

**Results:**

There was a general trend of adverse socio-demographic and lifestyle characteristics with higher h/d television-viewing, but trends in the opposite direction for work sitting. Television-viewing was associated with most biomarkers and associations were mediated by BMI: e.g. for each category increase in television-viewing, HDL-cholesterol in men was lower by 2.3% (95% CI: 1.5%, 3.2%) and, in BMI and diet adjusted analyses, by 1.6% (0.8%, 2.4%); for women, by 2.0% (1.2%, 2.9%) and 0.9% (0.1%, 1.6%) respectively. Few, weaker associations for work sitting were found, in men only: e.g. corresponding values for HDL-cholesterol were 1.2% (0.5%, 1.9%) and 0.9% (0.3%, 1.5%). Odds for metabolic syndrome were elevated by 82% and 33% respectively for men watching television or work sitting for ≥3 vs. 0–1 h/d.

**Conclusions:**

Associations with cardiovascular disease and diabetes biomarkers in mid-adulthood differed for television-viewing and work sitting. The role of sedentary behaviour may vary by leisure and work domains or the two indicators reflect differing associations with other disease-related influences.

## Introduction

Television-viewing/screen based entertainment (i.e. leisure-time television-viewing and computer use) has been found to be associated with raised mortality and cardiovascular disease (CVD) risk regardless of physical activity participation [1], [2], [3]. Moreover, television-viewing/screen-based entertainment has been found to be associated, independently of activity, with obesity [4], [5], dyslipidemia [4], [6], metabolic syndrome [7], [8], [9], [10], type 2 diabetes [11] and high blood pressure [4]. Such findings suggest that sedentary behaviour, i.e. activities that do not increase energy expenditure substantially above resting levels [12], may contribute to disease risk separately from physical activity, affecting health via independent pathways. Thus, sedentary behaviour and physical activity may be separate constructs. In the US, adults spend 55% of their waking hours in sedentary behaviour [13], and the time spent watching television increases year on year [14]. Given the ubiquitous and increasing nature of sedentary behaviours it is important to clarify their role in relation to health.

As the most frequently used measure of sedentary behaviour is television-viewing [15] most research has been done on sedentary behaviour in leisure. Although exceptions exist [16], [17], [18], [19], [20], there is a dearth of literature on other domains (e.g. occupational). There is uncertainty as to what television-viewing represents; it could be a general proxy for sedentary behaviour, or represent a combination of behaviours including dietary habits, as television-viewing is associated with increased energy intake [21], [22], [23], [24], possibly through snacking [25], [26]. Moreover, television-viewing may be the most common sedentary behaviour that adults engage in, but it occupies a small proportion of the waking day; most adults spend 7–10 h/d in sedentary behaviour, with work sitting often occupying much of this time [27]. To clarify whether sedentary behaviour is associated with disease-risk, it is important to replicate associations seen with television-viewing with other sedentary measures. Using data from the 1958 British Birth Cohort we aimed to establish whether (1) associations for television-viewing and work sitting with biomarkers for CVD/diabetes are consistent in direction and magnitude, and (2) television-viewing and work sitting have independent associations with CVD/diabetes biomarkers. Given uncertainties about what current indicators of sedentary behaviour (i.e. television-viewing) represent, we examined patterns of association with covariates for the two indicators to establish whether they are related similarly to other factors linked to disease-risk.

## Methods

Participants gave written informed consent to physical assessments and blood collection in the biomedical survey; ethical approval was given by South-East Multi-Centre Research Ethics Committee (ref: 01/1/44).

The 1958 cohort consists of 17,638 males and females followed-up since birth during one week in March 1958 across Britain [28]. A target sample (N = 11,971) were invited to participate in a biomedical survey at 44–45 y. By comparing the sample who took part in the survey to the surviving cohort on characteristics recorded at earlier sweeps, we determined that the 9,377 (78%) respondents were broadly representative of the total surviving cohort [29]. Our study uses data collected at 44–45 y, with some potential confounding variables collected earlier (detailed below). The available sample, in paid employment at 44–45 y, was 7,660, varying from 6140 to 7,613 depending on CVD/diabetes biomarker outcome.

### Outcomes

Nurses obtained all 44–45 y measurements using standardized protocols during home visits. Venous blood samples,obtained without prior fasting, were posted to a central laboratory. Total and high density lipoprotein (HDL)-cholesterol and triglyceride levels were analysed by autoanalyzer (Olympus AU640, Japan) using enzymatic methods. Low density lipoprotein (LDL)-cholesterol levels were calculated using the Friedewald formula [30], except when triglyceride levels were >4.5 mmol/L. Glycosylated haemoglobin (HbA1c) levels were measured using ion exchange high performance liquid chromatography. Fibrinogen was determined by the Clauss method, values >5.62 g/l (n = 8) were excluded due to potential distortion [31]. C-reactive protein (CRP) was assayed by nephelometry (Dade Behring) on citrated plasma after one thaw cycle, values >10 mg/l (n = 154) indicating acute inflammation were excluded [32]. After participants were seated for five minutes, blood pressure was measured three times (Omron 705CP, Tokyo, Japan). Mean diastolic and systolic blood pressure (DBP and SBP) was calculated using valid measures. Nurses observed packaging of currently prescribed medications, from which lipid-lowering, anti-hypertensive and oral anti-diabetic medications were identified. Hypertension was defined as SBP≥140 mmHg or DBP≥90 mmHg or medication for high blood pressure [33]. Metabolic syndrome was defined using a modified version of the ATPIII criteria [34]. We assumed that participants treated for diabetes, high blood pressure or dyslipidemia had HbA1c, blood pressure or triglycerides and HDL-cholesterol respectively at levels satisfying criteria for metabolic syndrome.

### Explanatory variables

Both television-viewing and work sitting were assessed using the previously validated EPIC-Norfolk physical activity questionnaire (EPAQ-2) [35], with minor modifications [36].

*Television-viewing:* participants reported average television-viewing in six categories, re-categorised to four levels: ‘0–1 h/d’, ‘1–2 h/d’, ‘2–3 h/d’ and ‘≥3 h/d’.

*Work Sitting:* was derived from the EPAQ-2 question “how many h/wk do you sit doing light work”, categorised into four levels, like television-viewing, from ‘0–1 h/d’ to ‘≥3 h/d’.

### Confounding and mediating factors

Frequency of leisure activities was derived from the EPAQ-2 questionnaire from a list of 35 activities and “free-text boxes”. Moderate-vigorous activity was defined as any activity that had a MET score≥3. We defined five categories for at least one moderate-vigorous activity with frequencies as follows: none in the last year, once a month or less, less than 3 times/wk, 3–5 times/wk and ≥6 times/wk. Self-reported smoking at 42 y (or 33 y if missing) was categorized as never, ex-smoker, or current smoker. Father's occupational class at birth (or 7 y if missing) was categorized using the Registrar General's Social Classification and grouped into: non-manual or manual/single mother. Occupational class of participants at 42 y (or 33 y if missing) was categorized similarly. Highest qualification at 42 y was grouped as none to O-levels or higher. Pre-existing conditions were identified as longstanding illness, disability or infirmity limiting daily activities at 42 y. Birth-weight, measured in ounces and pounds, was converted into kilograms. For women, hormone replacement therapy (HRT), oral contraceptive (OC) (both coded no, yes) and menopausal (pre-, peri-, or post-menopausal) status were ascertained at 45 y.

Diet and body mass index (BMI, kg/m^2^) were considered as potential mediators. At 42 y, participants reported food consumption frequency, including chips (three groups, <1 d/wk to 3+d/wk), sweets/chocolates, biscuits/cake and fruit (four groups,<1 d/wk to >1+times/d). Alcohol use at 45 y, using the Alcohol Use Disorders Identification Test questionnaire [37], was categorized into four groups from non- or light-drinker to very heavy drinker (>21 drinks/wk). We calculated BMI at 45 y, using height and weight, measured using Leicester portable stadiometers and Tanita solar scales, using self-reports if accurate measurements or consent for measurement were unavailable (weights n = 66; heights n = 50).

### Analyses

Geometric means are presented when biomarkers were skewed. To assess patterns of association for television-viewing and work sitting with factors linked to CVD/diabetes we investigated associations for the two behaviours with socio-demographic and lifestyle characteristics.

We assessed associations for the behaviours separately with each biomarker outcome using multiple linear regressions (continuous biomarkers) and logistic regressions (hypertension and metabolic syndrome). All continuous biomarkers were log-transformed and multiplied by 100: regression coefficients are interpreted as symmetric percentage differences in means [38]. Three models were fitted: first, unadjusted (Model 1); second, adjusted for socio-demographic and lifestyle characteristics (Model 2); third, to assess mediation, we adjusted for diet and BMI (Model 3). Analyses were repeated for Model 3 with separate adjustment for diet and BMI: generally the effect of diet was negligible (data not shown). In Model 3, we included three dietary indicators (chips, sweets/chocolate and alcohol consumption); we also undertook a sensitivity analysis in which additional factors (fruit, fish, biscuits/cake and pulse consumption) were included. Findings from sensitivity analyses were similar to those reported here (data not shown). In Models 2 and 3, to account for comorbidity, for all outcomes excluding lipids and metabolic syndrome, adjustment was made for total and HDL-cholesterol; for all outcomes excluding blood pressure and metabolic syndrome, adjustment was made for hypertensive status. Tests of deviation from linearity were performed in preliminary analyses of associations between each sedentary behaviour and biomarkers, using the likelihood ratio test. As a further check, we examined models where (for example) television-viewing was entered as a categorical or continuous term. There was no evidence of non-linearity and sedentary behaviour is presented as a continuous term. When associations were found for television-viewing and work sitting, we modelled both variables simultaneously to examine their independent association with CVD/diabetes biomarkers, with adjustments as above.

Ignoring or excluding participants on medication can bias associations [39], hence, corrections were made for those: on lipid-lowering drugs (n = 106, +25% for total cholesterol; +54% for LDL-cholesterol; +18% for triglycerides; −5% for HDL-cholesterol [40]), treated for high blood pressure (n = 333, +10 mmHg for DBP and SBP respectively [39]), and taking oral medication for type 2 diabetes (n = 82, +1% in absolute terms for HbA1c [41]). Those pregnant at 45 y (n = 2) were excluded from all analysis; those with type 1 diabetes were excluded from analyses of HbA1c (n = 42). 59% and 56% of women and men respectively had no missing data, while 23% of both genders had missing data on five or more variables. For educational achievement and OC use (for women) there was no missing data. Variables with most missing data were birthweight for women (8%) and smoking status for men (9%). To minimize data loss and potential bias due to missing information, missing covariates were imputed using multiple imputation chained equations and regression analyses were run across 10 imputed datasets.

As distributions differed for work sitting and television-viewing (Table 1) sensitivity analysis was performed with work sitting cut-points (h/d: 0, 0.1–2.6, 2.7–5.0 and >5.0) giving proportions in each category comparable to those for television-viewing. Results were similar to those reported here (data not shown). We assessed the effect of long-standing illness on associations of interest by excluding it from adjusted models, results were similar to those presented here (data not shown). We examined the impact of fieldwork factors, including examination month, time of day, recent food consumption, temperature, batch and sample delivery time to the laboratory. All showed negligible influences on associations of interest (data not shown). All regression analyses were gender specific.

**Table 1:** Characteristics of 45 y participants in paid employment and with a measure of television-viewing and sitting at work.

	Total		Women	Men
	N*	n(%) or mean (sd)	n (%) or mean (sd)	n (%) or mean (sd)
**Television-viewing (h/d)**	7,491			
0–1		1,015 (14.0)	557 (15.0)	494 (13.1)
1–2		2,684 (35.8)	1,314 (35.4)	1,370 (36.2)
2–3		2,217 (29.6)	1,115 (30.1)	1,102 (29.2)
3+		1,539 (20.5)	725 (19.5)	814 (21.5)
**Sitting at work (h/d)**	7,491			
0–1		2,464 (32.9)	1,478 (39.8)	986 (26.1)
1–2		796 (10.6)	385 (10.4)	411 (10.9)
2–3		1,031 (13.8)	554 (14.9)	477 (12.6)
3+		3,200 (42.7)	1,294 (34.9)	1,906 (50.4)
**Leisure-time moderate vigorous activity frequency**	7,491			
None		53 (0.71)	36 (0.97)	17 (0.45)
Infrequent		515 (6.87)	315 (8.49)	200 (5.29)
<3 times/wk		1,907 (25.46)	953 (25.68)	954 (25.24)
3–5 times/wk		2,148 (28.67)	1,060 (28.56)	1,088 (28.78)
≥6 times/wk		2,868 (38.29)	1,347 (36.30)	1,521 (40.24)
**CVD and diabetes biomarkers**				
Systolic blood pressure (mmHg)	7445	126.6 (16.4)	120.2 (15.4)	132.8 (14.9)
Diastolic blood pressure (mmHg)	7445	78.8 (10.8)	75.5 (10.2)	82.0 (10.4)
Total cholesterol (mmol/L)	6356	5.9 (1.1)	5.7 (1.0)	6.1 (1.1)
Triglycerides (mmol/L)†	6336	1.7 (1.7, 1.7)	1.3 (1.3, 1.4)	2.1 (2.0, 2.1)
HDL-cholesterol (mmol/L)	6342	1.6 (0.4)	1.7 (0.4)	1.4 (0.3)
LDL-cholesterol (mmol/L)	6009	3.4 (0.9)	3.3 (0.9)	3.6 (0.9)
HbA1c (%)†	6443	5.2 (5.2, 5.2)	5.1 (5.1, 5.2)	5.3 (5.2, 5.3)
Fibrinogen (g/litre)	6246	2.9 (0.6)	3.0 (0.6)	2.9 (0.6)
CRP (mg/litre)†	6107	0.9 (0.9, 0.9)	0.9 (0.9, 1.0)	0.9 (0.9, 0.9)
Metabolic syndrome (%)	6238	944 (15.1)	325 (10.7)	619 (19.4)
Hypertensive (%)	7445	1870 (25.1)	580 (15.8)	1290 (34.3)

Data not imputed and biomarker levels not corrected for medication.

CVD = cardiovascular disease; HDL = high-density lipoprotein; LDL = low-density lipoprotein; HbA1c = glycated haemoglobin; CRP = C-reactive protein.

*Total N varies due to variation in the amount of missing data.

† geometric means and 95%CIs.

## Results

Approximately 20% of participants watched television for ≥3 h/d, whereas 43% sat at work for ≥3 h/d (Table 1). Socio-demographic characteristics were clustered differently for television-viewing and work sitting. As television-viewing h/d increased, there was a trend of an increasing proportion with manual class in childhood and adulthood and lower education levels: e.g. the proportion of participants from a manual class at birth increased from 57% to 80% from 0–1 to ≥3 h/d television-viewing. Trends for work sitting were in the opposite direction: e.g. corresponding proportions were 78% to 65% for 0–1 to ≥3 h/d (Table 2). Distributions of life-style factors also varied for sedentary behaviours. For television-viewing there was an increasing trend with low fruit and high chips consumption, smoking, infrequent moderate-vigorous leisure activity and obesity from 0–1 to ≥3 h/d, but no trend for alcohol consumption. Trends in the opposite direction were seen with work sitting, for all factors except obesity and leisure activity. Neither television-viewing nor work sitting were associated with sweet/chocolate or cake/biscuit consumption (Table 2).

**Table 2:** Socio-demographic and lifestyle characteristics* of study participants at 45 y, stratified by sedentary behaviours.

		Television-viewing (h/d)				
	N**	0–1	1–2	2–3	3+	P for trend
		*Socio-demographic characteristics (%)*				
Manual social class at birth	7,284	57.1	66.0	72.8	80.3	<0.0001
Manual social class in adulthood	7,328	25.2	27.6	35.2	49.3	<0.0001
Education at 42 y (≤O-level)	7,491	34.8	41.0	53.2	66.3	<0.0001
	*Lifestyle characteristics (%)*					
Obese	7,479	15.9	20.0	26.9	29.7	<0.0001
Infrequent moderate-vigorous leisure activity†	7,491	5.4	5.9	7.3	12.5	<0.0001
Smoker	6,914	14.1	14.6	18.6	27.0	<0.0001
Very Heavy Drinker	7,466	7.3	7.5	9.4	12.3	0.93
Sweets/Chocolates (1+/d)	7,286	18.5	19.6	21.0	22.0	0.10
Chips (3+d/w)	7,286	3.8	6.7	7.6	13.0	<0.0001
Fruit (<1 d/wk)	7,286	12.1	13.3	17.6	27.4	<0.0001
Cakes/Biscuits (1+/d)	7,286	18.7	22.1	23.2	23.1	0.69

		Sitting at work (h/d)				
		0–1	1–2	2–3	3+	P for trend
		*Socio-demographic characteristics (%)*				
Manual social class at birth	7,284	77.6	67.1	67.5	65.0	<0.0001
Manual social class in adulthood	7,328	56.1	39.2	31.6	16.6	<0.0001
Education at 42 y (≤O-level)	7,491	61.5	42.3	43.9	42.5	<0.0001
	*Lifestyle characteristics (%)*					
Obese	7,479	24.6	20.3	22.2	23.8	0.73
Infrequent moderate-vigorous leisure activity†	7,491	8.3	7.7	6.0	7.5	0.19
Smoker	6,914	23.8	17.0	14.9	15.3	<0.0001
Very Heavy Drinker	7,466	8.3	9.6	7.9	9.9	<0.0001
Sweets/Chocolates (1+/d)	7,286	23.2	18.7	18.3	19.2	0.79
Chips (3+d/w)	7,286	9.3	7.7	6.5	7.3	<0.0001
Fruit (<1 d/wk)	7,286	21.2	16.6	14.9	15.3	0.001
Cakes/Biscuits (1+/d)	7,286	24.7	21.5	23.5	19.9	0.29

*One category shown in table.

**N for those in paid employment with a measure of television-viewing and sitting at work (genders combined); N varies because of missing data.

† none or infrequent (once a month or less) in last year.

**Figure 1:**
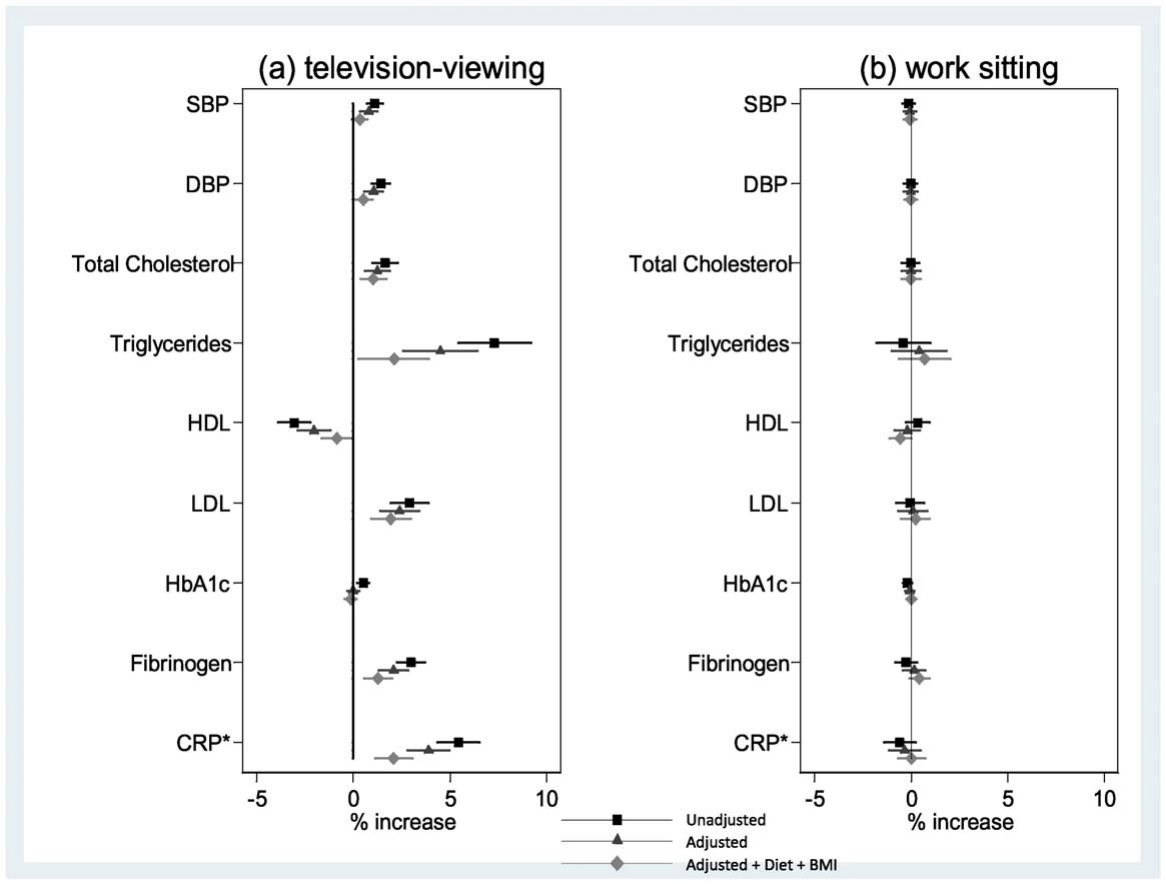
Mean % change (95% CI) in biomarker level, for women, per category increase in television-viewing (a) and sitting at work (b). Footnote: SBP = systolic blood pressure; DBP = diastolic blood pressure; HDL = high-density lipoprotein; LDL = low-density lipoprotein; HbA1c = glycated haemoglobin; CRP = C-reactive protein. *Quarter % change in CRP level per category increase in sedentary behaviour. Corresponding values for a % change in CRP per category increase in (a) television-viewing: 21.72% (95% CI: 17.42%, 26.02%), 15.56% (11.21%, 19.90%) and 8.39% (4.49%, 12.28%) and (b) sitting at work: −2.33% (−5.54%, 0.88%), −1.33% (−4.58%, 1.92%) and 0.13 (−2.74%, 3.00%) for “unadjusted”, “adjusted” and “adjusted+BMI+Diet” respectively. N varies from 3,752 to 3,037 due to variation in missing data. Model “Adjusted” includes: moderate-vigorous leisure activity frequency, smoking, social class at birth and in adulthood, education level, birth-weight, longstanding illness limiting daily activity, menopausal status, HRT and OC use; for total, HDL and LDL-cholesterol, triglycerides, HbA1c, fibrinogen and CRP additional adjustment for hypertension; for SBP, DBP, HbA1c,fibrinogen and CRP additional adjustment for total and HDL-cholesterol. Model “Adjusted+Diet+BMI” includes: all factors mentioned above plus consumption of chips, sweets/chocolates, alcohol, and BMI.

**Figure 2:**
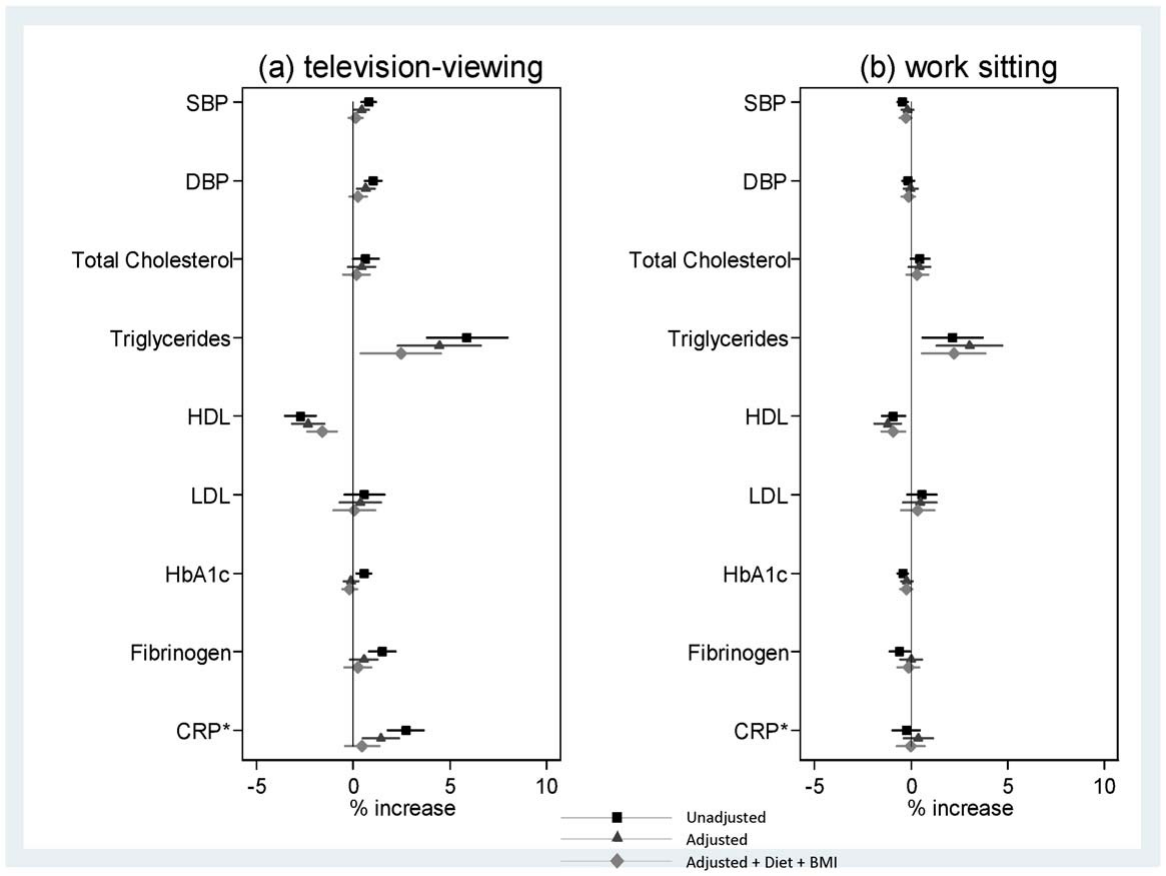
Mean % change (95% CI) in biomarker level, for men, per category increase in television-viewing (a) and sitting at work (b). Footnote: SBP = systolic blood pressure; DBP = diastolic blood pressure; HDL = high-density lipoprotein; LDL = low-density lipoprotein; HbA1c = glycated haemoglobin; CRP = C-reactive protein. *Quarter % change in CRP level per category increase in sedentary behaviour. Corresponding values for a % change in CRP per category increase in (a) television-viewing: 10.88% (95% CI: 7.2%, 14.57%), 5.73% (1.99%, 9.47%) and 1.83% (−1.72%, 5.37%) and (b) sitting at work: −1.01% (−3.80%, 1.78%), 1.47% (−1.57%, 4.51%) and −0.07% (−2.93%, 2.79%) for unadjusted”, “adjusted” and “adjusted+BMI+Diet” respectively. N varies from 3,861 to 3,024 due to variation in missing data. Model “Adjusted” includes: moderate-vigorous leisure activity frequency, smoking, social class at birth and in adulthood, education level, birth-weight and longstanding illness limiting daily activity; for total, HDL and LDL-cholesterol, triglycerides, HbA1c,fibrinogen and CRP additional adjustment for hypertension; for SBP, DBP, HbA1c, fibrinogen and CRP additional adjustment for total and HDL-cholesterol. Model “Adjusted+Diet+BMI” includes: all factors mentioned above plus consumption of chips, sweets/chocolates, alcohol, and BMI.

Associations for television-viewing and work sitting with biomarkers also differed. For women (Figure 1), higher television-viewing time (h/d) was associated with an adverse profile for all biomarkers except HbA1c, whereas work sitting was not related to any biomarker. In adjusted models, SBP, DBP, total and LDL-cholesterol, triglycerides, fibrinogen and CRP were higher by 0.8% (95% CI: 0.4%, 1.2%) to 15.6% (11.2%, 19.9%) and HDL-cholesterol lower by 2.0% (1.2%, 2.9%) per television-viewing category increase. In absolute terms, this corresponds to a difference in LDL-cholesterol, for example, of between 3.3 and 3.5 mmol/L for women viewing 0–1 and ≥3 h/d respectively. Associations were either fully mediated (SBP) or reduced after adjustment for BMI and diet (DBP, total-, HDL-, LDL-cholesterol, triglycerides, fibrinogen, CRP). For men (Figure 2), in adjusted models, neither behaviour was associated with total or LDL-cholesterol, HbA1c and fibrinogen, whilst SBP, DBP, triglycerides and CRP were higher by 0.4% (0.1%, 0.8%) to 5.7% (2.0%, 9.5%) and HDL-cholesterol lower by 2.3% (1.5%, 3.2%) per television-viewing category increase. After adjustment for diet and BMI, associations were fully mediated (SBP, DBP, CRP) or attenuated (triglycerides and HDL-cholesterol). Associations with work sitting were weaker (triglycerides higher by 3.0% (1.3%, 4.7%) and HDL-cholesterol lower by 1.2% (0.5%, 1.9%) per category increase in work sitting) or absent (Figure 2). In absolute terms, the estimated difference in HDL-cholesterol, for example, was between 1.40 and 1.30 mmol/L for men viewing for 0–1 and ≥3 h/d respectively; correspondingly 1.40 and 1.34 mmol/L for work sitting. In models of work sitting and television-viewing simultaneously (triglycerides and HDL-cholesterol in men), parameter estimates were little changed, indicating their independent associations: e.g. the adjusted estimate for triglycerides associated with each category increase in work sitting was 3.0% (1.3%, 4.7%) in Model 2 and 3.3% (1.6%, 5.0%) with further adjustment for television-viewing.

In women, television-viewing but not work sitting was associated with hypertension and metabolic syndrome (adjusted ORs 1.11 (1.01, 1.23) and 1.30 (1.15, 1.48)). Associations with hypertension were mediated by diet/BMI, while adjustment for diet had little effect on the association with metabolic syndrome (Table 3). In men, per category increase in television-viewing/work sitting, the adjusted OR for metabolic syndrome was 1.22 (1.11, 1.35), and 1.10 (1.02, 1.19) respectively; neither were attenuated by diet (Table 3). Thus, odds of metabolic syndrome were elevated by 82% and 33% for men with ≥3 h/d television-viewing or work sitting respectively, relative to those sedentary for 0–1 h/d. When both sedentary behaviours were modelled simultaneously for men, the ORs for the two behaviours were unchanged (data not shown).

**Table 3:** Associations (OR, 95% confidence intervals) for work time sitting and television-viewing with metabolic syndrome and hypertension.

	Metabolic Syndrome*				Hypertension			
	Odds Ratio† 95% Confidence Interval							
Women	Sitting at work		Television-watching		Sitting at work		Television-watching	
Unadjusted	0.96	0.88, 1.05	1.40	1.24, 1.58	1.04	0.97, 1.11	1.15	1.04, 1.26
Adjusted‡	1.01	0.92, 1.11	1.30	1.15, 1.48	1.04	0.96, 1.12	1.11	1.01, 1.23
Adjusted‡+Diet§	1.02	0.93, 1.12	1.30	1.14, 1.47	1.03	0.96, 1.11	1.09	0.98, 1.21
Adjusted‡+BMI	-		-		1.05	0.97, 1.13	1.05	0.95, 1.17
Adjusted‡+Diet§+BMI	-		-		1.04	0.96, 1.13	1.03	0.93, 1.14
**Men**								
Unadjusted	1.07	0.99, 1.14	1.27	1.16, 1.40	0.95	0.90, 1.00	1.12	1.04, 1.20
Adjusted‡	1.10	1.02, 1.19	1.22	1.11, 1.35	0.98	0.92, 1.04	1.06	0.99, 1.14
Adjusted‡+Diet§	1.11	1.02, 1.20	1.23	1.11, 1.35	0.97	0.92, 1.03	1.05	0.98, 1.14
Adjusted‡+BMI	-		-		0.96	0.90, 1.02	1.02	0.94, 1.10
Adjusted‡+Diet§+BMI	-		-		0.96	0.90, 1.02	1.01	0.93, 1.09

*No adjustment for BMI, as central adiposity is a component of metabolic syndrome.

† OR for an increase per category of television-viewing/sitting at work.

‡ Adjusted for: moderate-vigorous leisure activity frequency, smoking, social class at birth and in adulthood, education level, birth-weight and longstanding illness limiting daily activity; for women, additional adjustment for menopausal status, HRT and OC use; for hypertension, additional adjustment for total and HDL-cholesterol.

§ Consumption of chips and sweets/chocolates at 42 yr and alcohol at 45 yr.

## Discussion

In this large population-based study, a main finding is the different associations of television-viewing and work sitting with biomarkers for CVD/diabetes in mid-adulthood. Firstly, higher levels of television-viewing had adverse associations with almost all biomarkers whereas, associations with work sitting were sparser and weaker. Secondly, differences in associations with CVD/diabetes biomarkers for television-viewing and work sitting are perhaps unsurprising given our finding that these indicators have different patterns of association with other factors linked to CVD/diabetes. Generally, there was a trend of adverse socio-demographic and lifestyle characteristics with increasing television-viewing, but a trend in the opposite direction for work sitting, suggesting that associations may be confounded in different ways. Yet, for the few associations observed for work sitting the direction of association was similar to that for television-viewing and, when examined simultaneously, both showed independent relationships. Thirdly, all associations for television-viewing and work sitting with biomarkers were mediated by BMI; for metabolic syndrome, diet did not mediate associations with either behaviour.

Study strengths include the availability of information on (i) two major domains of sedentary behaviour in our daily lives (work and leisure), whilst most other studies use only television-viewing (leisure domain) [15], [27], (ii) a range of CVD/diabetes biomarkers, including inflammatory markers, (iii) measures of BMI and diet, enabling an examination of their mediating role, (iv) several socio-economic and life-style characteristics, some pertaining to earlier life-stages, permitting multiple adjustments, and (v) a nationwide cohort such that our findings apply to the general population, although restricted to those in paid employment, as imposed by a study involving both leisure and work-based sedentary indicators. Sample attrition had occurred over follow-up, although previous work has shown 44–45 y respondents to be broadly representative of the surviving cohort [29] and further loss due to missing information was handled by multiple imputation. Lipids were measured using non-fasted blood: while total and HDL-cholesterol are little affected, triglyceride levels are lower after fasting and vary by fasting duration. Fasting and non-fasting triglycerides are correlated [42], and a meta-analysis found no variation in results for triglycerides by fasting status [43]. Thus, non-fasting lipids are likely to be acceptable for our analyses. As plasma glucose was not measured we rely on HbA1c as a marker of long-term glucose homeostasis.

To some extent, there is temporal ordering of exposures and outcomes; although they were measured cross-sectionally in mid-adulthood, participants reported usual (sedentary) behaviour in the last year. The use of television-viewing as an indicator of leisure-time sedentary behaviour, in this and other studies, may be inadequate if individuals spend large amounts of time in other sedentary actives (e.g. computer use). While, in this cohort of middle-aged adults, over 75% used computers in leisure for <1 h/d, future studies, particularly in younger populations, may need to consider a broader range of behaviours from this domain. Self-reported data on sedentary behaviour might be considered a study limitation. However, from objectively measured (i.e. accelerometer) data alone it is not possible to identify the domains in which behaviour occurs, which we show here to be of potential importance. Hence, alternative measures including questionnaires may be informative [44]. It has been argued that both domain-specific and overall (objective) measures of sedentary time are desirable [45]. In our study, time spent in both sedentary behaviours was calculated from questionnaires, but different formats were used: for occupational sitting participants estimated their total h/wk sitting doing light work, whereas for television-viewing they responded using pre-defined categories (h/d). Further, repeatability of the EPAQ-2 questionnaire for work activity (of which work sitting is a sub-component) was found to be weaker than that for television-viewing [35]. Hence, it is possible that television-viewing and work sitting were not measured with the same precision. Although analyses were adjusted for leisure time activity, we did not adjust for moderate-vigorous activity at work. The latter is highly correlated (negatively) with sitting time at work and thus, taking account of this factor is likely to represent over-adjustment. We were unable to take account of activity in other domains (e.g. home), although we assessed the impact of transport to work (always using motorised transport vs. cycling or walking) in sensitivity analysis and results were little changed (data not presented).

Whether diet and BMI act as mediating or confounding factors is uncertain, yet sequential additions to models allowed an assessment of their individual and combined impact on associations of interest, and demonstrated noteworthy effects of BMI and negligible effects of diet. To limit the number of highly correlated dietary variables in our analysis, we considered only three indicators in our models. Sensitivity analysis findings that included additional dietary factors were similar to those reported here. However, assessment of diet was prior to the measures of exposures and outcomes and this may contribute to its negligible effect on our associations of interest. Similarly, other covariates were measured prior to exposure and outcome assessment; however this is unlikely to affect our results as such factors as educational attainment and occupational class tend to be stable over our 3 yr period in mid-adulthood. We adjusted for long-standing illness limiting daily activities because, potentially, these could affect both sedentary behaviours and biomarker outcomes. However, conditions such as high blood pressure might be identified as limiting illness. In sensitivity analysis, we excluded long-standing illness from adjusted models and results were almost identical to those presented here. Despite adjustment for multiple factors, it is possible that there is unmeasured confounding.

We have used two sedentary indicators from different domains. If television-viewing and work sitting were both capturing effects of sedentary behaviour only, we expect both indicators to have broadly similar associations with CVD/diabetes biomarkers. Yet, fewer and weaker associations were found for work sitting than for television-viewing. This finding is consistent with previous studies of sedentary behaviour from different domains in women [17], [18]. In the Nurses' Health Study [17], both television-viewing and work sitting were associated with obesity and diabetes, but television-viewing associations were stronger. Similarly for epithelial ovarian cancer, television-viewing associations were stronger than those for work sitting [18]. There are several possible reasons for discrepant findings for these sedentary indicators. Firstly, there are differences in metabolic expenditure, with a lower expenditure for television-viewing than sitting doing office work [46]. Hence, stronger associations for television-viewing may reflect the lower energy expenditure of this behaviour. Secondly, the constellation of socio-demographic and lifestyle factors associated with television-viewing, both past and present, suggests that it may represent a broad summary index for factors associated with adverse health. Conversely, work sitting was associated with favourable socio-demographic and lifestyle factors and had only weak associations with fewer biomarkers. Despite multiple adjustments to minimise confounding, the net effect of differing associations with other CVD/diabetes-related factors could be that television-viewing overestimates and/or work sitting underestimates true associations of sedentary behaviours. Given that most studies have focused on leisure-time recreation [47], current understanding of the role of sedentary lifestyles may be limited because of possible bias associated with this indicator.

Pathways through which sedentary behaviour affects CVD/diabetes risk are yet to be fully investigated, but evidence to date suggests that it may operate separately from activity [6], [48], [49], [50]. For example, a two-fold (women) and 48% (men) increase in odds of metabolic syndrome was reported for Australian adults watching television for >14 vs. ≤7 h/wk, independent of activity [48]. Similarly, we found higher odds of metabolic syndrome greater than twofold (women) and by 82% (men), for those watching television for ≥3 h/d vs. 0–1 h/d, after accounting for activity. Our finding of no association between sedentary behaviour and HbA1c, adds to the literature on sedentary behaviour and diabetes, for which there is limited evidence [27]. For some biomarkers, a major pathway through which sedentary behaviour might affect health is via adiposity, although few studies address this possibility. Specifically, many studies of television-viewing do not compare models with and without diet/BMI, and therefore potential pathways through diet/BMI cannot be assessed. For example, the EPIC-Norfolk study, found adverse associations between television-viewing and SBP, DBP, triglycerides, total, HDL- and LDL-cholesterol after accounting for BMI [6] (not all of which were observed here). We are unable to assess the mediating role of adiposity across our study and the EPIC-Norfolk cohort without information from the latter on the reduction in effect size due to adjustment for BMI. Similar to our findings, Fung et al. [49] found an attenuation of the television-viewing and HDL-cholesterol association in men after allowing for BMI, although we did not see their finding of an association with LDL-cholesterol (men only) that was little affected by BMI. Our study suggests that BMI mediates the association between television-viewing and CVD related outcomes. However, given evidence from our study and others [6], [49], additional pathways may operate for some markers, particularly lipids (e.g. HDL-cholesterol). Notably, it has been suggested elsewhere that a possible pathway may be through the effect of inactivity on lipoprotein lipase (LPL) that regulates plasma triglycerides and HDL cholesterol. Although evidence is limited in humans, LPL function and triglyceride clearance by skeletal muscle was lower in rats that were inactive compared to those who were able to stand/amble [51].

In conclusion, associations with biomarkers in mid-adulthood differed for television-viewing and work sitting, suggesting that the role of sedentary behaviour varies by domain or that the two indicators reflect differing associations with other disease-related influences. Yet some associations were common to both sedentary indicators, albeit stronger for television-viewing, and with evidence of mediation by BMI. These findings add to growing evidence that sedentary behaviour, separately from activity, influences health even though uncertainties remain regarding underlying pathways. Given the ubiquitous and increasing nature of sedentary behaviours [13], [14] further investigation is warranted to clarify the impact of behaviours from different domains.
